# The Optimal Design of the Press Roller to Improve the Winding Molding Quality of Heat Insulation

**DOI:** 10.3390/ma17081769

**Published:** 2024-04-12

**Authors:** Weichao Zhang, Zengxuan Hou, Hongli Li, Kaiyin Chen

**Affiliations:** State Key Laboratory of High-Performance Precision Manufacturing, School of Mechanical Engineering, Dalian University of Technology, Dalian 116024, China; zwc@mail.dlut.edu.cn (W.Z.); lihongli@mail.dlut.edu.cn (H.L.); kaiyinc@mail.dlut.edu.cn (K.C.)

**Keywords:** press roller, pressure distribution, heat insulation, winding molding, finite element model

## Abstract

In the heat insulation winding molding process of solid rocket motors, the pressure applied by the press roller directly affects the quality of the winding molding. Insufficient pressure can result in poor bonding quality and may cause defects. This paper aims to provide an optimal design of the press roller to improve the winding molding quality of the heat insulation. The effect of the cylindrical press roller on the pressure distribution was analyzed using the elastic foundation model and a finite element (FE) model, which was assessed by Hertz theory. Subsequently, the press roller was optimized to an elliptical concave design. The effect of the radius of the elliptical concave press roller on the pressure distribution was analyzed. A comparison of the effect of the elliptical concave press roller and the cylindrical press roller on the pressure distribution was conducted using the FE model. The results show pressure uniformity is significantly improved when the elliptical concave press roller is employed on the mandrel with the smallest radius. Additionally, the elliptical concave press roller increases the pressure at the edge of the tape, which reduces the risk of lifted edges and, thereby, improves the winding molding quality of the heat insulation.

## 1. Introduction

**Figure 1:**
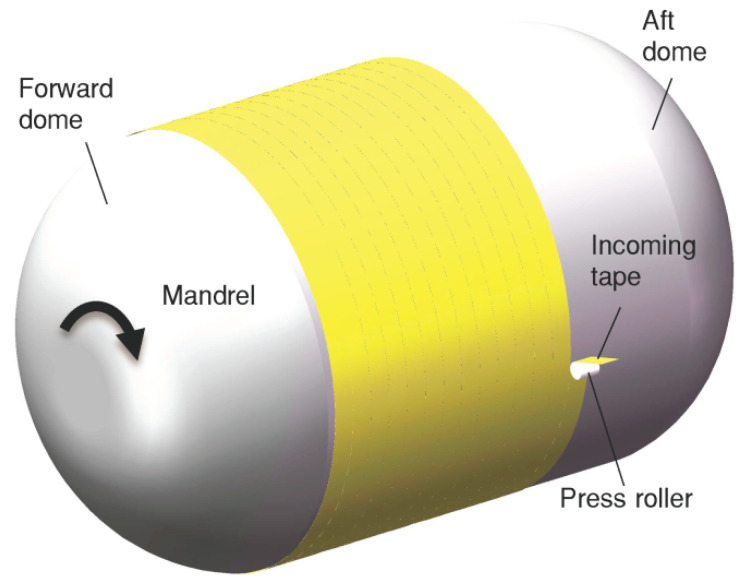
Heat insulation winding molding process.

Internal heat insulation in a solid rocket motor is a layer of heat-barrier, ablation-resistant material between the internal surface of the case and the propellant. The primary function of the heat insulation is to prevent the case from reaching temperatures that endanger its structural integrity [1] (p. 1). The fabrication techniques for the heat insulation encompass various methods, such as the manual lay-up process, the molding process, and the winding molding process [2,3,4,5]. The winding molding process, a relatively new technique, is particularly suited for manufacturing large solid rocket motors. This method not only shortens the manufacturing cycle, but also ensures the consistent quality of the heat insulation when compared with the manual lay-up process. In the heat insulation winding molding process, each tape is transported to the press roller independently and then wound onto the mandrel surface or previous layers at a certain winding angle and velocity, as shown in Figure 1 [5]. The main role of the press roller is to apply a specific pressure to bond the tape to the mandrel surfaces or previous layers.

In composite automated placement, the press roller directly affects pressure distribution [6,7,8,9]. Several researchers have analyzed the effect of the roller on pressure distribution and sought to improve layup quality. For example, Gonzalez Ojeda et al. [10] established a mapping relationship between force, contact area, and pressure through FE simulation for automated fiber placement (AFP). Cheng et al. [11] studied the contact characteristics of the roller and the morphological change in the prepreg under the roller during composite automated placement. In terms of press roller optimization, Sonmez et al. [12] analyzed the relationship between process parameters and the bonding behavior of the thermoplastic composite laminate by FE analysis. Their findings suggest that consolidation is more effectively achieved with larger roller diameters. Bakhshi et al. [13] examined the effect of the stiffness and structure of the roller on the layup quality of AFP. Jiang et al. [14] investigated the effect of the roller on the pressure distribution in AFP numerically and experimentally, and optimized the structure of the roller. However, most of these studies focus on optimizing the materials and dimensions of conventional cylindrical press rollers, with fewer research efforts dedicated to improving layup quality through the optimization of roller shapes.

Similar to composite automated placement, the winding molding quality of the heat insulation is also affected by the press roller [15]. The pressure applied by the roller is even more important in the heat insulation winding molding process than composite automated placement because it directly affects the final bonding quality of the heat insulation. The advantage of conventional cylindrical press rollers is their relatively simple structure. However, in the heat insulation winding molding process, if a conventional cylindrical press roller is employed, the distance between the roller’s profile and the mandrel’s profile (or the profile of the heat insulation) varies along the tape width direction. At the edge of the tape, the distance is larger and the pressure is smaller, which affects the winding molding quality and may even cause defects such as lifted edges. There are few reports on how to improve the winding molding quality of the heat insulation through the optimal design of the roller.

The method presented in this paper is aimed at providing an optimal design of the press roller to improve the winding molding quality of the heat insulation. The effect of the cylindrical press roller on the pressure distribution is analyzed using the elastic foundation model. An FE model, assessed by Hertz theory, is used to simulate the contact between the roller and the tape. The results show that the cylindrical press roller results in a relatively nonuniform pressure distribution and may cause lifted edges. The press roller is then optimized to an elliptical concave design. The effect of the radius of the elliptical concave press roller on the pressure distribution is analyzed to optimize the design parameter. Finally, a comparison of the effect of the elliptical concave press roller and the cylindrical press roller on the pressure distribution is conducted using the FE models.

## 2. Theoretical Analysis of Pressure Distribution

**Figure 2:**
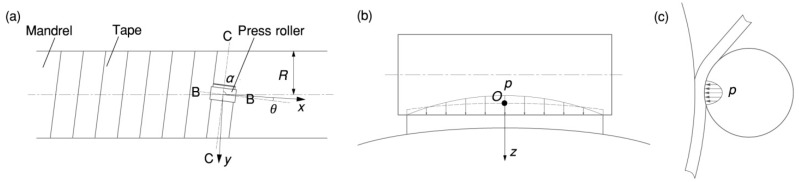
Contact between roller and tape: (**a**) hoop winding; (**b**) contact in the longitudinal section B-B; (**c**) contact in the cross-section C-C.

The fabrication techniques we employed for the heat insulation are as follows: the heat insulation of the forward and aft domes uses the molding process, while the heat insulation of the cylinder section employs the winding molding process. In the winding molding process, the roller is pressed into the tape to a specific depth to apply pressure [16], as shown in Figure 2. The longitudinal section B-B intersects with the center of the roller and is perpendicular to the winding direction. The cross-section C-C provides an alternative perspective. In Figure 2, the winding angle is represented by α, and the pressure is represented by p.

The elastic foundation model was used to calculate the pressure distribution [17,18,19]. The calculations are based on the following assumptions:

- The characteristic length of the contact area is assumed to be comparable to or larger than the thickness of the heat insulation.
- Given their high elastic modulus, both the press roller and the mandrel are considered rigid, i.e., the elastic modulus of the materials of these elements is assumed to be infinitely large.
- There is no shear between the adjacent elements of the foundation (the heat insulation).

Designate the initial contact point as the origin of the rectangular coordinate system. In this system, the x-y plane corresponds to the common tangent plane at the intersection of the two surfaces, while the z-axis aligns with the common normal, extending positively into the heat insulation. According to the elastic foundation model, the normal displacement of the heat insulation at any point (x, y) within the contact area can be obtained [20].

(1) $$\delta =\delta_{0}-Ax^{2}-By^{2}$$

where $\delta_{0}$ is the penetration at the origin of the coordinate system. The coefficients A and B are calculated as follows:

(2) $$A=\frac{1}{4}\left(\frac{1}{\left(R+t\right)}+\frac{1}{r}\right)-\frac{1}{4}{\left(\frac{1}{{\left(R+t\right)}^{2}}+\frac{1}{r^{2}}-\frac{2\cos(2\alpha )}{\left(R+t\right)r}\right)}^{\frac{1}{2}}$$

(3) $$B=\frac{1}{4}\left(\frac{1}{\left(R+t\right)}+\frac{1}{r}\right)+\frac{1}{4}{\left(\frac{1}{{\left(R+t\right)}^{2}}+\frac{1}{r^{2}}-\frac{2\cos(2\alpha )}{\left(R+t\right)r}\right)}^{\frac{1}{2}}$$

where R is the radius of the mandrel, t is the thickness of the heat insulation, and r is the radius of the roller. The winding angle α is calculated with Equation (4).

(4) $$\cos\alpha =\frac{W}{2\pi (R+t-h)}$$

where W is the width of the tape, h is the thickness of the tape.

The pressure is expressed as:

(5) $$p=K\frac{\delta }{t}$$

(6) $$K=\frac{E\left(1-\nu \right)}{\left(1+\nu \right)\left(1-2\nu \right)}$$

where E and ν are the elastic modulus and Poisson’s ratio of the tape, respectively.

**Figure 3:**
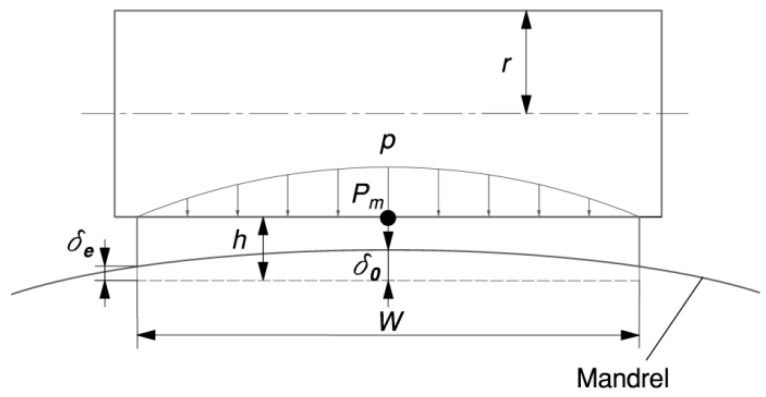
Effect of the cylindrical press roller on tape deformation in the longitudinal section B-B for t = h.

According to Equation (1), the relationship between the deformation $\delta_{0}$ in the middle (see point *P_m_* in Figure 3) of the tape and the deformation ${\;\delta }_{e}$ at the edge of the tape can be obtained.

(7) $$\delta_{e}=\delta_{0}-\frac{W^{2}}{4}\left(A\cos^{2}\theta +B\sin^{2}\theta \right)$$

(8) $$\tan\left(2\theta \right)=\frac{r\sin\left(2\alpha \right)}{\left(R+t\right)-r\cos\left(2\alpha \right)}$$

where θ is the angle between the x-axis and the axis of the press roller.

The deformation $\delta_{0}$ in the middle of the tape is maximal, while the deformation $\delta_{e}$ at the edge of the tape is minimal. According to Equation (5), $\delta_{0}$ and $\delta_{e}$ correspond to the maximum pressure $p_{max}$ and the minimum pressure $p_{min}$, respectively. To fulfill the minimum pressure requirement $p_{r}$ for bonding quality, $p_{min}$ should be larger than or equal to $p_{r}$. Due to the presence of the winding angle and the non-compliance of the rigid cylindrical press roller with the mandrel, it is difficult to ensure the pressure at the edge of the tape, which may result in lifted edges.

## 3. Simulation of Pressure Distribution

In the heat insulation winding molding process, the friction force between the roller and the tape is small, and it has little effect on normal pressure. As a result, the dynamic winding process can be simplified into a static one [13,14].

### 3.1. Finite Element Model

A 3D FE model of the contact between the roller and the tape was established using Abaqus/CAE. The mandrels have radii of 240 mm, 400 mm, and 1000 mm, respectively. The cylindrical press roller has a length of 102 mm and a radius of 40 mm. The tape has a thickness of 0.8 mm and a width of 100 mm. The material of the tape is uncured EPDM (ethylene propylene diene monomer) rubber. Its stress–strain behavior exhibits linearity when the deformation is less than 5%. Consequently, a constant elastic modulus and Poisson’s ratio are employed to characterize the rubber’s elastic deformation behavior [21,22]. The material parameters of each part are presented in Table 1. The elastic modulus of the tape was estimated to be 0.5 MPa [23]. Given that the tape is made of uncured rubber, its behavior is close to incompressible [24]. Consequently, the Poisson’s ratio of the tape was estimated to be 0.49.

**Figure 4:**
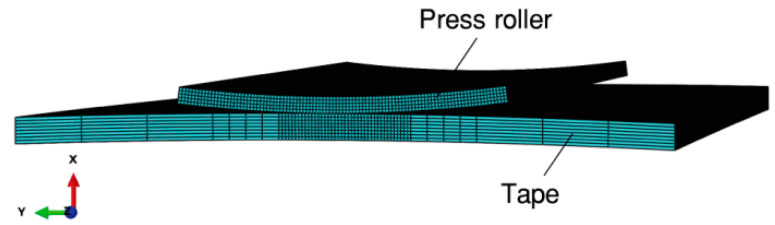
Finite element model of the contact between the roller and the tape.

To improve the calculation efficiency, only one layer (t = 0.8 mm) is modeled. The mandrel is omitted, and the cylindrical press roller is modeled as an annular sector body with a thickness of 0.5 mm, and is constrained as a “rigid body”. The modifications of the roller are less important in the simulation due to its much greater stiffness compared to the tape [25]. Finite element discretization of the tape is performed using C3D8IH. The press roller is discretized using C3D8I. The meshes for the contact problem are carefully designed with fine elements placed along the interface between the roller and the tape [26]. Figure 4 represents the mesh for one of the simulations. Given that the press roller is defined as a rigid body using a rigid body constraint in Abaqus/CAE, it requires meshing [14].

The lower surface of the tape is fixed as it is bonded to the mandrel. The translational and rotational degrees of freedom of the press roller are constrained at the rigid body reference point defined at the center of the roller. The press roller is constrained to move only in the x-direction, with pressure applied through a specified displacement of the roller. A frictionless hard contact is defined between the press roller surface and the tape to simulate the contact. Finally, the contact problem was solved utilizing the standard Abaqus algorithm. In the simulation results, contact pressure (CPRESS) within the tape’s contact area is extracted for the analysis of pressure distribution.

**Figure 5:**
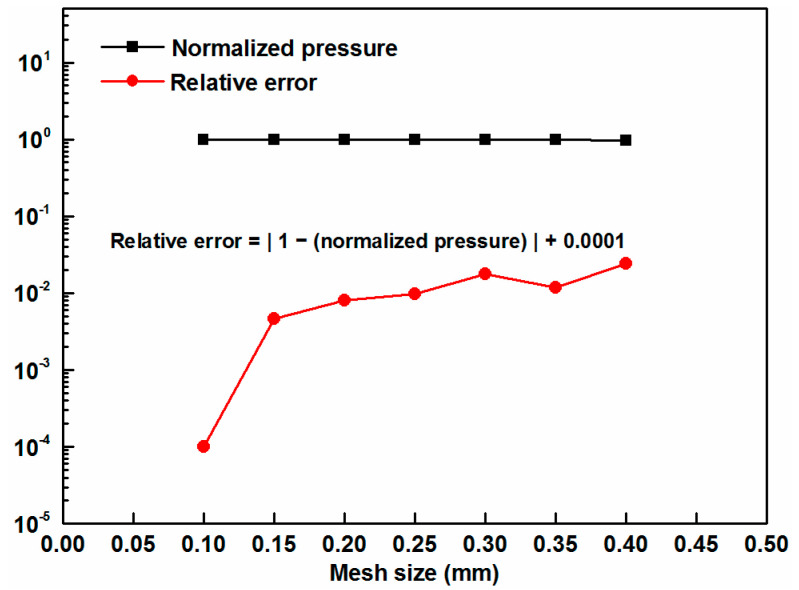
Mesh sensitivity analysis of the simulation model (a constant of 0.0001 was added to all relative errors to allow for zero values on the logarithmic scale).

Pressure distribution is the primary parameter of interest here. Hence, convergence analysis on the maximum pressure was conducted to ensure the independence of the results from mesh size [13]. Element sizes from 0.4 mm to 0.1 mm were tried for the tape as a convergence study, the results of which are presented in Figure 5.

In this figure, the pressure value at each data point is normalized by the pressure value obtained using the smallest mesh size. A mesh size of 0.1 mm was determined to be sufficiently fine for obtaining accurate results.

### 3.2. Assessment of the Simulation Model Using Hertz Theory

To assess the simulation model, a comparison with Hertz theory was performed.

Hertz theory is based on the half-space theory [27]. It provides a good approximation for small strains and non-conforming solid contact problems. According to Hertz theory, the shape of the contact area between the roller and the tape is elliptical, centered on the initial contact point with the semi-axes a and b. The pressure distribution is semi-ellipsoidal with a maximum pressure $p_{0}$ at the center. By using a rectangular coordinate system (the same as in Section 2), the Hertz pressure distribution is given by Equation (9):

(9) $$p=p_{0}\sqrt{1-\frac{x^{2}}{a^{2}}-\frac{y^{2}}{b^{2}}}$$

The semi-axes a, b, and the maximum pressure $p_{0}$ is calculated as follows [28] (p. 36):

(10) $$\left\{\begin{array}{c} a=\sqrt{\frac{\delta_{0}}{Ae^{2}}\left[1-\frac{E\left(e\right)}{K\left(e\right)}\right]}\\ \begin{array}{l} b=a\sqrt{1-e^{2}}\\ p_{0}=\frac{E^{*}\delta_{0}}{bK\left(e\right)} \end{array} \end{array}\right.$$

in which e is the eccentricity of the contact area, K(e) is the complete elliptic integral of the first kind, E(e) is the complete elliptic integral of the second kind, and $E^{*}$ is the equivalent modulus:

(11) $$\left\{\begin{array}{c} \frac{1}{K\left(e\right)-E\left(e\right)}\left[\frac{E\left(e\right)}{1-e^{2}}-K\left(e\right)\right]=\frac{B}{A}\\ \begin{array}{l} K\left(e\right)=\int_{0}^{\frac{\pi }{2}}\frac{d\theta }{\sqrt{1-e^{2}\cos^{2}\theta }}\\ E\left(e\right)=\int_{0}^{\frac{\pi }{2}}\sqrt{1-e^{2}\cos^{2}\theta }d\theta \end{array} \end{array}\right.$$

(12) $$E^{*}={\left(\frac{1-\nu^{2}}{E}\right)}^{-1}$$

where coefficients A and B are calculated according to Equations (2) and (3).

When the compressive displacement ($\delta_{0}$) is large, the contact between the roller and the tape does not satisfy the contact conditions of Hertz theory. This is because the characteristic length of the contact area is comparable to or larger than the thickness of the heat insulation. To adhere to Hertz theory, two options are available: reducing the compressive displacement or increasing the thickness of the heat insulation. Reducing only the compressive displacement will result in a decrease in the contact half-width as well. To ensure the accuracy of the simulation results, the edge size of the contact area element should be correspondingly reduced [25]. However, this will significantly increase the computational scale. Increasing only the thickness of the heat insulation will also lead to a large computational scale. This paper adopts a method of increasing the heat insulation thickness and reducing the compressive displacement while maintaining element sizes consistent with the FE model of one layer.

**Figure 6:**
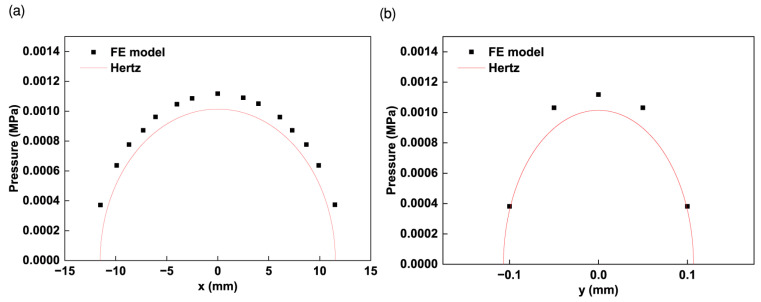
Comparison of the pressure distribution estimated by the FE model and Hertz theory for R = 240 mm, t = 20 mm, and $\delta_{0}$ = 0.001 mm: (**a**) pressure distribution along the semi-axis a; (**b**) pressure distribution along the semi-axis b.

Figure 6 shows the assessment results of the FE model (R = 240 mm, t = 20 mm, $\delta_{0}$ = 0.001 mm).

Although the results of the pressure distribution obtained from the FE model and Hertz theory are not in perfect agreement, the simulation model is still a reasonable tool. This discrepancy can be attributed to the fact that for a compressive displacement of 0.001 mm, the minimum edge size (0.05 mm) of the element is approximately half the semi-axes b (b = 0.107 mm). Some previous tests showed that when the edge size of the element is much smaller than the size of the contact area, the simulation results tend to align more closely with the predictions of Hertz theory [25]. Given that the FE model of one layer shares the same mesh as this model, it is also considered a reasonable and suitable tool for simulating the contact between the roller and the tape.

### 3.3. Simulation Results of Pressure Distribution

**Figure 7:**
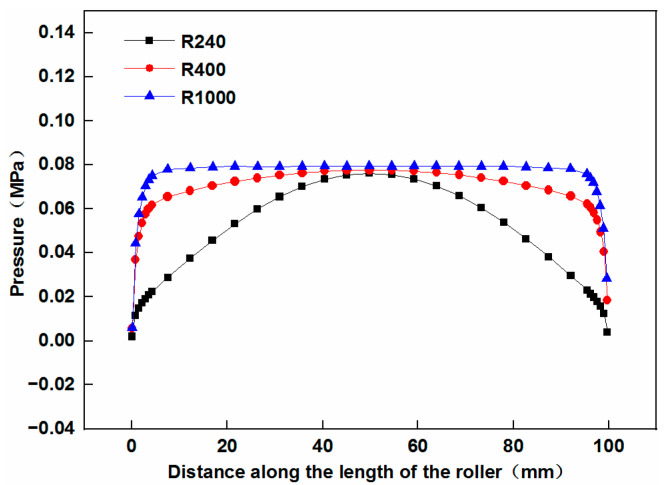
Effect of the cylindrical press roller on the pressure distribution in the longitudinal section B-B.

Figure 7 shows the pressure distribution in the longitudinal section B-B under a compressive displacement of 0.03 mm.

It is clear that when the cylindrical press roller is employed on the mandrel with the smallest radius, the uniformity of the pressure distribution in the longitudinal section B-B is relatively low, and the pressure at the edge of the tape is minimal. As the radius of the mandrel increases, the uniformity of the pressure distribution also increases. However, the pressure at the edge of the tape remains minimal, making it difficult to ensure bonding quality and may cause lifted edges.

## 4. Optimization of Press Roller Design

In the heat insulation winding molding process, the uniformity of the pressure distribution is affected by two main components: the press roller and the mandrel radius. The mandrel radius is predetermined before the fabrication process. When the cylindrical press roller is employed, the uniformity of the pressure distribution is relatively low and may cause lifted edges. Therefore, to improve the winding molding quality of the heat insulation, optimization of the press roller is necessary.

### 4.1. Shape Optimization for the Press Roller

**Figure 8:**
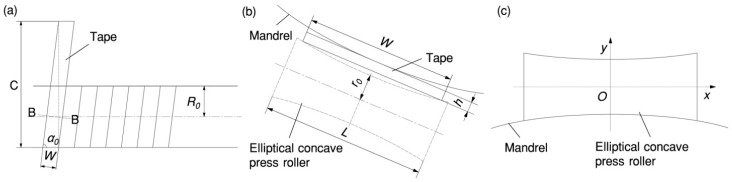
Shape design of the elliptical concave press roller: (**a**) unwinding the tape along the winding direction; (**b**) longitudinal section B-B view; (**c**) elliptical concave press roller.

The shape of the press roller was optimized to an elliptical concave surface [15]. The method for designing the shape of the elliptical concave press roller is as follows:

1. As shown in Figure 8a,b, the standard equation for the ellipse of the mandrel in the longitudinal section B-B is:
  (13) $$\frac{x^{2}\cos^{2}\alpha_{0}}{R_{0}^{2}}+\frac{y^{2}}{R_{0}^{2}}=1$$
  where $R_{0}$ is the radius of the smallest mandrel, and $cos\alpha_{0}=W/C=W/(2\pi R_{0})$.
2. Construct the elliptical rotating surface of the roller. As shown in Figure 8b, the generatrix of the roller can be obtained by translating the contour of the mandrel.

In the coordinate system shown in Figure 8c, the generatrix equation for the roller is:

(14) $$\left\{\begin{array}{c} \frac{x^{2}\cos^{2}\alpha_{0}}{R_{0}^{2}}+\frac{{\left(y+R_{0}+r_{0}\right)}^{2}}{R_{0}^{2}}=1\\ -\frac{L}{2}\leq x\leq \frac{L}{2}\\ -\left(r_{0}+R_{0}\right)\leq y \end{array}\right.$$

where L is the length of the roller and $r_{0}$ is the radius of the central cross-sectional circle of the elliptical concave press roller. The equation for the elliptical concave surface of the roller can be obtained.

(15) $$\left\{\begin{array}{c} y^{2}+z^{2}={\left(-\sqrt{R_{0}^{2}-x^{2}\cos^{2}\alpha_{0}}+R_{0}+r_{0}\right)}^{2}\\ \frac{-L}{2}\leq x\leq \frac{L}{2} \end{array}\right.$$

### 4.2. Effect of Elliptical Concave Press Roller Radius on Pressure Distribution

In Section 4.1, we outlined the design of the elliptical concave press roller. The length of the roller can be determined by the width of the tape, while the radius of its central cross-sectional circle is yet to be defined. To determine and optimize this design parameter, the effect of the radius of the central cross-sectional circle of the elliptical concave press roller on the pressure distribution is analyzed.

Simulation models of one layer were established using the method described above. The mandrels used in these models have a radius of 240 mm. The elliptical concave press rollers have a length of 102 mm, and the radii of their central cross-sectional circles are 20 mm, 40 mm, and 70 mm, respectively. The pressure at point *P_m_* of the tape is regulated to 0.076 MPa by adjusting the compressive displacement.

**Figure 9:**
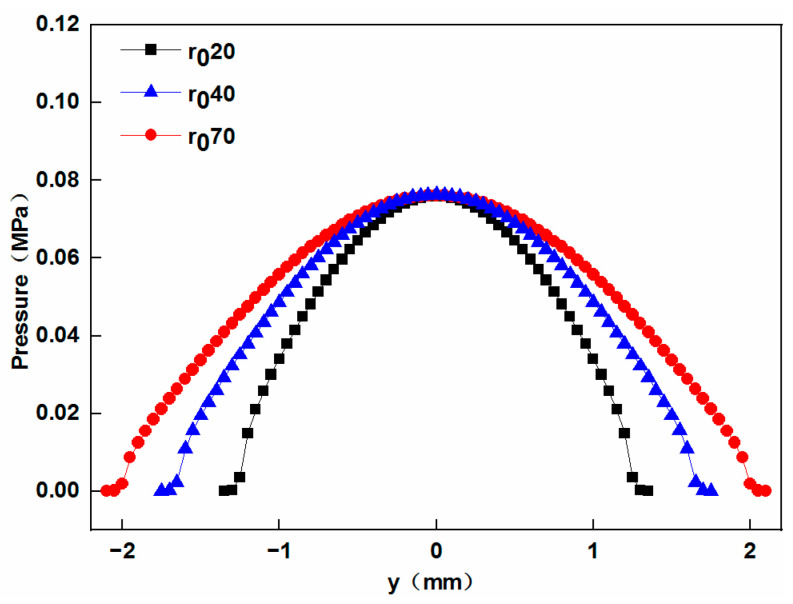
Effect of the radius of the central cross-sectional circle of the elliptical concave press roller on the pressure distribution along the width of contact.

The simulation results of the pressure distribution along the width of contact are shown in Figure 9.

It can be found that larger-radius elliptical concave press rollers exhibit a wider contact width and longer dwell time (defined as the average time for each tape to be subjected to pressure), which indicates a higher bonding quality of the heat insulation. These findings align with the conclusions drawn by Sonmez et al. [12]. They found that in the process of thermoplastic composite tape placement, a decrease in the press roller radius leads to a reduction in dwell time, potentially resulting in incomplete bonding.

**Figure 10:**
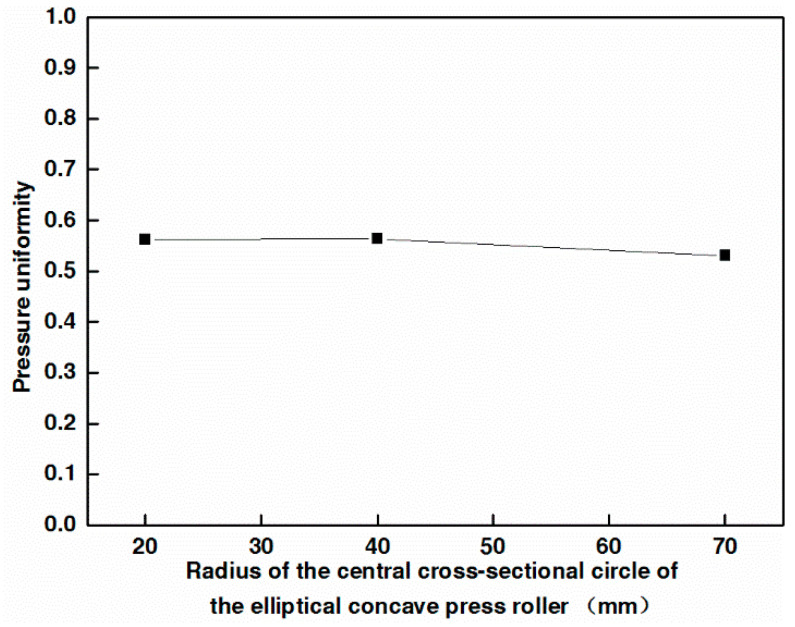
Effect of the radius of the central cross-sectional circle of the elliptical concave press roller on pressure uniformity.

Figure 10 shows the effect of the radius of the central cross-sectional circle of the elliptical concave press roller on pressure uniformity. The pressure uniformity is calculated according to Equation (16) [29].

(16) $$u=1-\sqrt{\frac{1}{n}\overset{n}{\underset{i=1}{\Sigma }}{\left(\frac{p_{i}-p_{aver}}{p_{aver}}\right)}^{2}}$$

where n is the number of nodes, $p_{i}$ is the contact pressure value at the node i, $p_{aver}=\frac{1}{n}\sum_{i=1}^{n}p_{i}$, and $p_{aver}$ is the average contact pressure value of all nodes. A larger value of Equation (16) indicates a more uniform pressure distribution, while a smaller value suggests less uniformity.

As shown in Figure 10, the pressure uniformity essentially remains constant with the increase in the radius of the central cross-sectional circle of the elliptical concave press roller, disregarding the slight decrease (less than 6%) in the pressure uniformity.

This finding contradicts the conclusion that an increase in the outer diameter of the flexible roller’s rubber cover leads to a more uniform pressure distribution [14]. The reason for this contradiction is that the deformability of the flexible roller’s rubber cover increases as its outer diameter enlarges. This increased deformability enables it to adapt more effectively to molds, thereby improving pressure uniformity. In contrast, increasing the radius of the rigid roller does not enhance its adaptability to the mandrel.

### 4.3. Comparative Analysis of the Effect of the Elliptical Concave Press Roller and the Cylindrical Press Roller on Pressure Distribution

**Figure 11:**
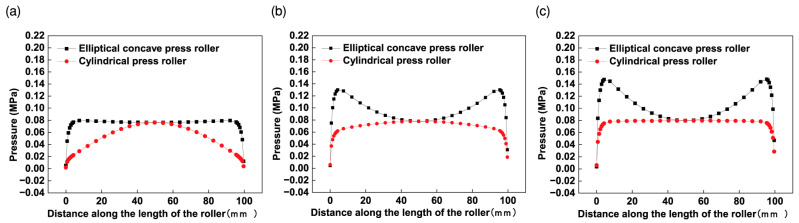
Effect of the elliptical concave press roller and the cylindrical press roller on the pressure distribution for three different radius mandrels: (**a**) R = 240 mm; (**b**) R = 400 mm; (**c**) R = 1000 mm.

A comparison of the effect of the elliptical concave press roller and the cylindrical press roller on the pressure distribution was conducted using the FE models. The dimensions of the cylindrical press roller and the mandrels, as detailed in Section 3.1, are used in this study. The elliptical concave press roller used has a length of 102 mm, and the radius of its central cross-sectional circle is 40 mm. A compressive displacement of 0.03 mm is set for the simulations. Figure 11 presents the effect of both the elliptical concave press roller and the cylindrical press roller on the pressure distribution in the longitudinal section B-B.

The simulation results presented in Figure 11 are similar to the findings of Jiang et al. [14]. They conducted both numerical and experimental investigations on the pressure distribution when the cylindrical flexible roller contacts prepreg tows on three types of molds in AFP. The three types of molds are: flat, concave and convex molds, both of the latter with a curvature radius of 900 mm. Their results showed the pressure distribution curve along the length of the roller on the convex mold is essentially convex, while that on the concave mold is essentially concave. In Figure 11, when the cylindrical press roller is employed, the pressure distribution curve is convex. This is attributed to the similarity between the contact of the cylindrical press roller with the cylindrical mandrel (convex) in this study and the contact of the cylindrical press roller with the convex mold. In Figure 11, when the elliptical concave press roller is employed, the pressure distribution curve is concave. This is due to the similarity between the contact of the elliptical concave press roller with the cylindrical mold in this study and the contact of the cylindrical press roller with the concave mold.

**Figure 12:**
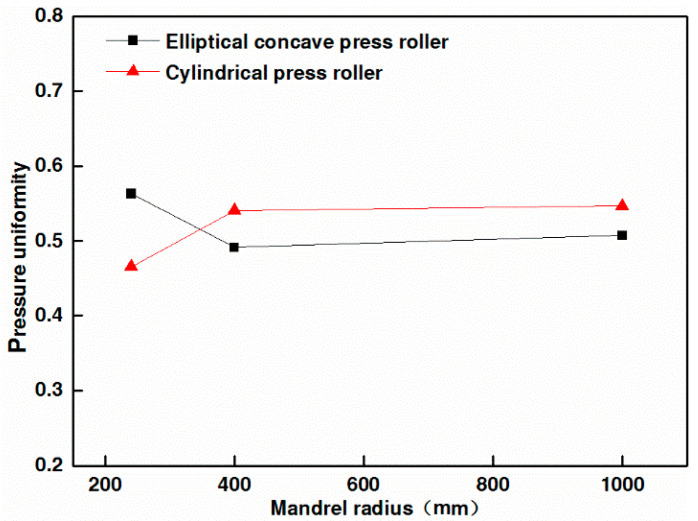
Effect of the elliptical concave press roller and the cylindrical press roller on the pressure uniformity.

Figure 12 shows the effect of the elliptical concave press roller and the cylindrical press roller on the pressure uniformity. As shown in Figure 11a and Figure 12, the employment of the elliptical concave press roller on the mandrel with the smallest radius significantly improves the pressure uniformity in comparison to the employment of the cylindrical press roller. Figure 11b,c and Figure 12 show that the pressure uniformity decreases slightly when the elliptical concave press roller is employed on other mandrels compared to the cylindrical press roller. However, the pressure at the edge of the tape is larger than that at point *P_m_* of the tape when the elliptical concave press roller is employed. Consequently, the elliptical concave press roller can ensure the bonding quality of the heat insulation, provided the pressure at point *P_m_* meets the bonding quality requirements. The simulation results confirm the effectiveness of the elliptical concave press roller in improving the winding molding quality of the heat insulation. Moreover, a single elliptical concave press roller can be employed to wind mandrels of different radii, thus increasing manufacturing efficiency.

## 5. Conclusions

This paper presents an optimal design of the press roller to improve the winding molding quality of the heat insulation. The effect of the cylindrical press roller on the pressure distribution was investigated using the elastic foundation model and an FE model, which was assessed by Hertz theory. The results show that the cylindrical press roller results in a relatively nonuniform pressure distribution and minimal pressure at the edge of the tape, making it difficult to ensure bonding quality and may cause lifted edges. Subsequently, the press roller was optimized to an elliptical concave design. The effect of the radius of the central cross-sectional circle of the elliptical concave press roller on the pressure distribution was analyzed to optimize the design parameter. Elliptical concave press rollers with larger radii demonstrate a wider contact width and longer dwell time, indicating a higher bonding quality of the heat insulation. The radius of the central cross-sectional circle of the elliptical concave press roller has little effect on the pressure uniformity. A comparison of the pressure distribution between the elliptical concave press roller and the cylindrical press roller was conducted using the FE model. The results show that the pressure uniformity is significantly improved when the elliptical concave press roller is employed on the mandrel with the smallest radius. Additionally, the elliptical concave press roller increases the pressure at the edge of the tape, which reduces the risk of lifted edges. The simulation results confirm the effectiveness of the elliptical concave press roller in improving the winding molding quality of the heat insulation. Furthermore, a single elliptical concave press roller can be employed to wind mandrels of different radii, thereby boosting manufacturing efficiency.

Most notably, this is the first study to our knowledge to investigate the application of elliptical concave press rollers in the heat insulation winding molding process. This paper introduces a novel method for assessing FE models using Hertz theory. This method can be extended to the assessment of contact FE models that involve flexible press rollers, coatings, articular cartilage, and so forth. However, some limitations are worth noting. Due to the neglect of shear between adjacent elements of the foundation by the elastic foundation model, there is a certain discrepancy between its pressure values and the simulation results. Future work should therefore focus on the theoretical and experimental study of the contact between the elliptical concave press roller and the tape to provide theoretical support for the pressure control of the heat insulation winding molding process.

## Figures and Tables

**Table 1:** Material parameters of the mandrel, the press roller, and the tape.

	Elastic Modulus (MPa)	Poisson’s Ratio
Mandrel	1,000,000	0.1
Press roller	200,000	0.3
Tape	0.5	0.49

## Data Availability

All data, models, or code that support the conclusions of this study are available from the corresponding author upon reasonable request.
